# Dubin–Johnson syndrome and intrahepatic cholestasis of pregnancy in a Sri Lankan family: a case report

**DOI:** 10.1186/s13104-017-2811-6

**Published:** 2017-09-18

**Authors:** Grace Angeline Malarnangai Kularatnam, Hewa Warawitage Dilanthi, Dinesha Maduri Vidanapathirana, Subashini Jayasena, Eresha Jasinge, Ginige Nalika Nirmalenede Silva, Kirinda Liyana Arachchige Manoj Sanjeeva Liyanarachchi, Pujitha Wickramasinghe, Manjit Singh Devgun, Veronique Barbu, Olivier Lascols

**Affiliations:** 1 Department of Chemical Pathology, Lady Ridgeway Hospital for Children, Colombo, Sri Lanka; 2 Lady Ridgeway Hospital for Children, Colombo, Sri Lanka; 3 Department of Paediatrics, Faculty of Medicine, University of Colombo, Colombo, Sri Lanka; 4 Clinical Laboratories, Department of Biochemistry, Wishaw General Hospital, Wishaw, Lanarkshire ML2 0DP UK; 5 Laboratoire Commun de Biologie et de Génétique Moléculaires, Hôpital Saint-Antoine, 184, rue du Faubourg Saint-Antoine, 75012 Paris, France

**Keywords:** Dubin–Johnson syndrome, Intrahepatic cholestasis of pregnancy, Case report, Conjugated hyperbilirubinemia, Coproporphyrin isomers, Liver pigmentation, Bile acids, ABCC2, ABCB11

## Abstract

**Background:**

Dubin–Johnson syndrome and intrahepatic cholestasis of pregnancy are rare chronic liver disorders. Dubin–Johnson syndrome may manifest as conjugated hyperbilirubinemia, darkly pigmented liver, presence of abnormal pigment in the parenchyma of hepatocytes and abnormal distribution of the coproporphyrin isomers I and III in the urine. Intrahepatic cholestatic jaundice of pregnancy presents as pruritus, abnormal liver biochemistry and increased serum bile acids.

**Case presentation:**

A Sri Lankan girl presented with recurrent episodes of jaundice. She had conjugated hyperbilirubinaemia with diffuse, coarse brown pigments in the hepatocytes. Urine coproporphyrin examination suggested Dubin–Johnson syndrome. Genetic studies confirmed missense homozygous variant p.Trp709Arg in the ATP-binding cassette sub-family C member 2 gene *ABCC2* that encodes the Multidrug resistance-associated protein 2 that causes Dubin–Johnson syndrome. The gene study of the mother revealed the same missense variant in *ABCC2*/MRP2 but with a heterozygous status, and in addition a homozygous missense variant p.Val444Ala in the ATP-binding cassette, sub-family B member 11 gene *ABCB11* that encodes the bile salt export pump.

**Conclusion:**

Dubin–Johnson syndrome should be considered when the common causes for conjugated hyperbilirubinaemia have been excluded, and patient has an increased percentage of direct bilirubin relative to total bilirubin concentration. Its early diagnosis prevents repeated hospital admissions and investigations. Knowledge of a well known homozygous variant in *ABCB11* gene could help in the management of pregnancy.

## Background

Dubin Johnson syndrome (DJS) is a rare autosomal recessive disorder characterised by chronic or intermittent conjugated hyperbilirubinemia due to absence of functional Multidrug Resistance-associated Protein 2 (MRP2) [1]. The *ABCC2* gene that encodes the MRP2 is in chromosome 10q24. The MRP2 protein is located in the canalicular membrane of hepatocytes and also at the apical membranes of cholangiocytes and enterocytes. It has been shown that mutation in ABCC2 gene results in impaired secretion of conjugated bilirubin and other anionic conjugates into bile [2]. Jaundice from intrahepatic cholestatic jaundice of pregnancy (ICP) is a common liver disease during the second and third trimester that can arise from mutations in the *ABCB11* gene encoding the bile salt export pump (BSEP) [3]. We present, herein, a genetic study in a family with a history of jaundice.

## Case presentation

A three and a half year-old girl presented to a Base Hospital in Sri Lanka, with fever, jaundice and dark yellow coloured urine of 10 days duration. She was treated with an Ayurvedic Medicine (a form of alternative medicine practised in South Asian countries) for the same complaint few weeks prior to this episode which improved the symptoms transiently. All previous episodes of mild jaundice and febrile illnesses had resolved spontaneously and did not raise any particular parental concern.

The girl was born to non-consanguineous parents and had a healthy elder sister. Her maternal grandmother has history of intermittent jaundice and passage of dark urine. There is no other known history of similar condition in the family. She was born by normal vaginal delivery and her birth weight was 3.92 kg. There were no antenatal or perinatal complications, and no record of neonatal jaundice. Also, there is no history of blood or blood product transfusion and no history of prolonged intake of any medication.

On physical examination, she was not pale but moderately icteric with the liver palpable 4 cm below the right costal margin and the spleen palpable 2 cm below the left costal margin. There were no peripheral stigmata of chronic liver disease. Also, there was no evidence of Kayser–Fleischer rings indicating that hepatolenticular degeneration (Wilson’s disease) is unlikely. The ultrasound scan of abdomen revealed evidence of hepatomegaly with a smooth organ outline and a normal hepatic echo texture. Hepatic and portal veins were normal. Hepatobiliary-iminodiacetic-acid cholescintigraphy (HIDA) scan did not visualise the intra- or extra-hepatic bile ducts, thus indicating the possibility of presence of DJS.

Laboratory data during the acute episode showed total bilirubin (TBIL) of 102 µmol/L (3–20) with direct fraction (DBIL) of 67 µmol/L (<3), that is, DBIL/TBIL at 67% [4]. Alanine transaminase (ALT), aspartate transaminase (AST), alkaline phosphatase (ALP) and gamma-glutamyltransferase (GGT) were 58 U/L (10–40), 87 U/L (9–48), 218 U/L (80–480) and 11 U/L (2–30) respectively. Coagulation profile was normal. White blood cell count was normal and the Haemoglobin was 10.5 g/dL (11.0–13.5). There was no evidence of active haemolysis in the blood picture. Bilirubin was detected in urine. The serology was negative for viral hepatitis A, B and Epstein–Barr virus. Anti-nuclear antibody, anti-smooth muscle antibody and anti-mitochondrial antibody were negative. 24-h urinary copper excretion after pencillamine challenge was normal.

**Fig. 1:**
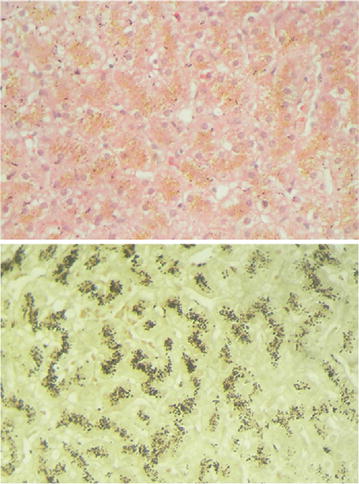
Top image—enlarged hepatocytes in patient’s liver biopsy show coarse brown pigment granules. Bottom image—liver biopsy show pigments positivity with the melanin stain

Under the light microscope the liver biopsy revealed enlarged hepatocytes with coarse brown pigments in the cytoplasm. Hepatocytes stained positive with melanin resulting in an insoluble black precipitate and with Periodic acid-Schiff stain (PAS) depicting brown granular pigments from glycogen, mucin and basement membrane (Fig. 1). The liver tissue stained negative with Perls Prussian blue, indicating absence of hepatic iron overload. Also, there was no chelation of tissue’s parenchymal substance with Rubeanic acid, thus ruling against the presence of copper deposition and confirming our clinical observation of absent Kayser–Fleischer rings. Her urine porphyrin:creatinine ratio was 61 nmol/mmol (<40) with coproporphyrin isomer I of 81% of total urinary coproporphyrin excretion.

**Fig. 2:**
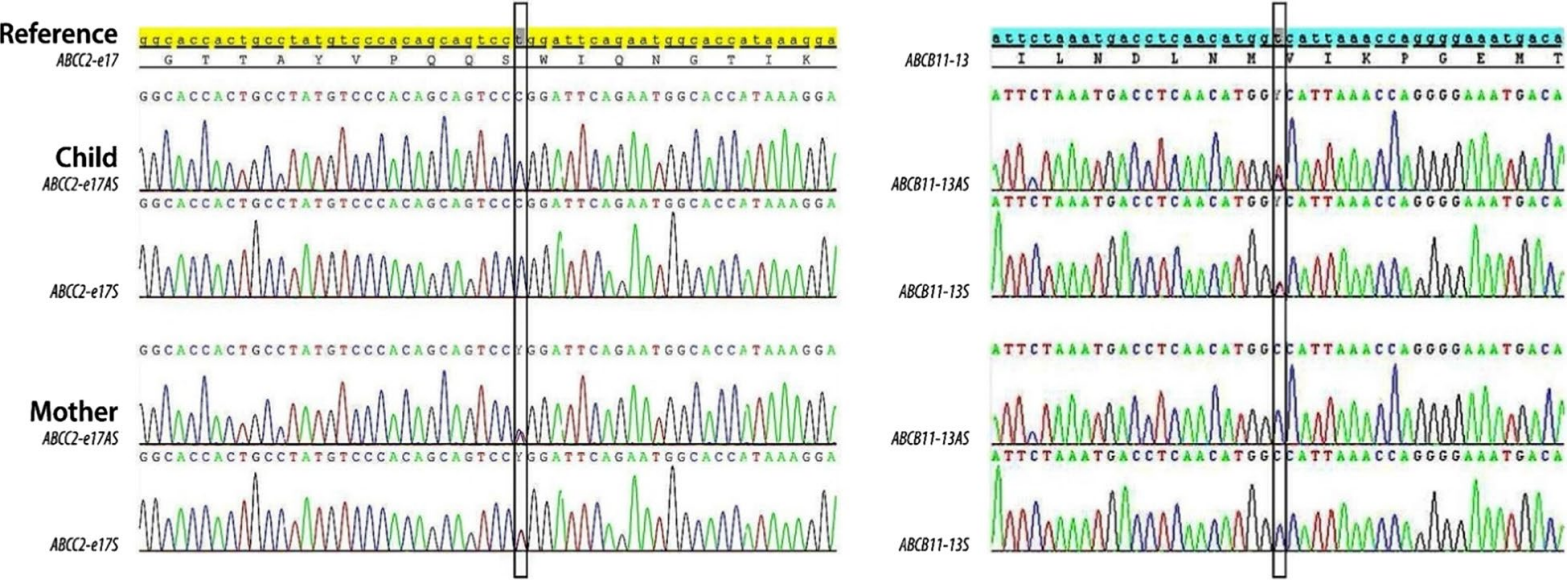
Sanger sequence chromatograms of exons 17 and 13 of *ABCC2* and *ABCB11* genes, respectively, for the child and mother. The genomic DNA was from peripheral blood using Puregene Kit (QIAGEN), and primers’ sequences for polymerase chain reaction were designed as follows: *ABCC2_17F 5′TGGGGCTTTTAATGGTGAAG3′. ABCC2_17R 5′GTGTAGTTCTTCACCACCATC3′. ABCB11_13F 5′ACTTCTTGGTCATGGCTCTCAG3. ABCB11_13R 5′AGCGTGTCCCATCAATTCAG3′*

Genetic studies revealed that she is homozygous for the ABCC2/MRP2 gene (Fig. 2), resulting from missense mutation variant p.Trp709Arg (exon 17, c.2125T>C). The genomic DNA was prepared from peripheral blood using standard methods (Puregene Kit, QIAGEN France SAS, 3 avenue du Canada, LP 809, 91974 COURTABOEUF CEDEX). Primers’ sequences and polymerase chain reaction (PCR) products were designed as follows:

- ABCC2_ 17F 5′TGGGGCTTTTAATGGTGAAG3′.
- ABCC2_17R 5′GTGTAGTTCTTCACCACCATC3′.
- ABCB11_ 13F 5′ACTTCTTGGTCATGGCTCTCAG3′.
- ABCB11_ 13R 5′AGCGTGTCCCATCAATTCAG3′.

The whole ABCC2/MRP2 gene sequencing has been previously described [2]. The girl had no significant mutation in the following canalicular transport genes [5]: ABCB11/BSEP, the gene deficiency identified for progressive familial intrahepatic cholestasis 2 (PFIC2) and benign recurrent intrahepatic cholestasis type 2 (BRIC2); the phosphatidylcholine transporter ATP binding cassette Subfamily B Member 4 (ABCB4) encoding the multidrug resistance 3 protein (MDR3), that causes progressive familial intrahepatic cholestasis type 3 (PFIC3); the phospholipid-flippase ATPase phospholipid transporting, class 1, type 8B, member 1 (ATP8B1/F1C1), that encodes a member of the P-type cation transport ATPase family, resulting in the progressive familial intrahepatic cholestasis type 1 (PFIC1) and biallelic mutations in the benign recurrent intrahepatic cholestasis type 1 (BRIC1).

Child’s mother (28 years old) was mildly jaundiced but did not have hepatosplenomegaly. There was no history of cholestasis during pregnancy, and it is uncertain whether she had pruritus and or raised bile acids and hepatic transaminases during pregnancy. Mother’s TBIL was 37 µmol/L (5–21), DBIL 6 µmol/L (<3.4) and DBIL/TBIL at 16%. Other liver function tests were normal. Her urine porphyrin:creatinine ratio was 11 nmol/mmol (<40) and coproporphyrin isomer I of 39% of total coproporphyrin. Genetic studies revealed the mother to be heterozygous for the ABCC2/MRP2 gene, due to missense mutation variant p.Trp709Arg. Moreover, an additional well known missense variant p.Val444Ala in the gene ABCB11/BSEP was found in the homozygous state (Fig. 2). It is considered p.Trp709Arg is a harmful and disease causing variant, located in the intracytoplasmic loop 6 (IC6) of MRP2 protein, within Walker A motif of the nucleotide-binding domain 1 (NBD1) [6]. Similarly, p.Val444Ala variant gives susceptibility to hormonal cholestasis or ICP [7]. In the mother, no significant mutation was detected in genes ABCB4/MDR3, and ATP8B1/FIC1.

The young girl remains well with intermittent jaundice precipitated by febrile illnesses. The child’s sister is clinically well and her investigations did not reveal any clinical or biochemical abnormality. For social reasons, we could not clinically investigate the father.

## Discussion

Dubin–Johnson syndrome has been described worldwide but it is highly prevalent among Iranian Jews [8]. Patients are rarely diagnosed during the neonatal period [8]. Patients usually present with recurrent episodes of jaundice precipitated by infection, pregnancy, oral contraceptives or drugs [6] and have high percentage of DBIL/TBIL. Because of resource limitation, DJS and ICP cases remain undiscovered or not fully investigated and its prevalence in Sri Lanka is therefore unknown. Nevertheless, in the absence of any septic condition, ultrasonically detectable liver abnormality or potentially interfering medications, diagnosis of DJS should be suspected in patients exhibiting pruritus, isolated conjugated hyperbilirubinaemia with increased percentage of DBIL/TBIL or disordered liver transaminases [4].

Abnormal distribution of the coproporphyrin isomers I and III in the urine is a characteristic feature of DJS. The urinary excretion rate of corproporphyrin isomer I is >80% of the total urinary coproporphyrin while total urinary excretion of coproporphyrin is normal or slightly elevated [9]. Urinary coproporphyrin isomer I excretion is approximately 40% in carriers [9].

There are around 18 different mutations reported in *ABCC2* gene including splice-site, missense, nonsense mutations and deletions [1]. Missense p.Trp709Arg variant is reported in DJS patients [10].

Prevalence of ICP is around 1% in Northern Europe, and is more common in Scandinavian and Chilean ancestry [11]. It is rare in Asia, but this may be because of low case detection rate. *ABCB11* gene encodes ATP-binding cassette transporter (BSEP) which is expressed in the hepatocyte canalicular membrane [12]. Mutations in this gene are responsible for reduced biliary secretion of bile salt which leads to decreased bile flow and accumulation of bile salts inside the hepatocytes, thus resulting spectrum of cholestatic disease. p.Val444Ala variant has been reported to be correlated with cholestasis [13] and chronic hepatitis C virus infection [14]. ICP can potentially be monitored using total bile acid measurement, and managed using ursodeoxycholic acid [3].

It is quite clear from this case presentation, that how important laboratory services are in both making a diagnosis and also managing the patients. Therefore, efforts should be made to establish a centralised laboratory for testing which would help to provide timely and effective medical care for patients presenting with hyperbilirubinaemia such as DJS and ICP cases.

## Conclusion

In cholestatic hepatobiliary disorders early establishment of diagnosis is important to avoid unnecessary evaluations and prescribing of medication. DJS is a benign disorder that does not require treatment and, in each specific case, DBIL/TBIL% should be monitored in order to rule out possibility of co-existence of unconjugated hyperbilirubinaemia. ICP is either very rare or very rarely investigated due to scarcity of resources in the field of biochemical and molecular genetics in the South East Asian regions. We have highlighted the gene mutations present in these possibly the first reported cases of DJS and ICP in the Indian subcontinent. Establishment of a centralised laboratory service is highly desirable and necessary to provide effective medical care for such conditions. More importantly and without prejudice, in mutational disorders, such a centralised facility would provide a highly persuasive tool to convince patients not to waste precious resources in seeking answers and cures from alternative forms of medicine. Provision of such a service would be a powerful instrument that is sure to add to the wealth of medical knowledge of the region.

## Abbreviations

ABCB4: ATP binding cassette, subfamily B, member 4
ABCB11: ATP-binding cassette, sub-family B, member 11
ABCC2: ATP-binding cassette, sub-family C, member 2
ALP: alkaline phosphatase
ALT: alanine transaminase
AST: aspartate transaminase
ATP8B1: ATPase phospholipid transporting, class 1, type 8B, member 1
BSEP: bile salt export pump
BRIC1: benign recurrent intrahepatic cholestasis type 1
BRIC2: benign recurrent intrahepatic cholestasis type 2
DBIL: direct (conjugated) bilirubin
DJS: Dubin–Johnson syndrome
GGT: gamma-glutamyl transferase
HIDA: hepatobiliary-iminodiacetic-acid cholescintigraphy
IC6: intracytoplasmic loop 6
ICP: intrahepatic cholestasis of pregnancy
MDR3: multi-drug resistance protein 3
MRP2: multi-drug resistance protein 2
NBD1: nucleotide-binding domain 1
PAS: periodic acid schiff stain
PCR: polymerase chain reaction
PFIC1: progressive familial intrahepatic cholestasis type 1
PFIC2: progressive familial intrahepatic cholestasis type 2
PFIC3: progressive familial intrahepatic cholestasis type 3
TBIL: total bilirubin
