# Correction to: Dubin–Johnson syndrome and intrahepatic cholestasis of pregnancy in a Sri Lankan family: a case report

**DOI:** 10.1186/s13104-017-2815-2

**Published:** 2017-10-05

**Authors:** Grace Angeline Malarnangai Kularatnam, Hewa Dilanthi Warawitage, Dinesha Maduri Vidanapathirana, Subashini Jayasena, Eresha Jasinge, Ginige Nalika Nirmalene de Silva, Kirinda Liyana Arachchige Manoj Sanjeeva Liyanarachchi, Pujitha Wickramasinghe, Manjit Singh Devgun, Veronique Barbu, Olivier Lascols

**Affiliations:** 1 Department of Chemical Pathology, Lady Ridgeway Hospital for Children, Colombo, Sri Lanka; 2 Lady Ridgeway Hospital for Children, Colombo, Sri Lanka; 3 Department of Paediatrics, Faculty of Medicine, University of Colombo, Colombo, Sri Lanka; 4 Clinical Laboratories, Department of Biochemistry, Wishaw General Hospital, Wishaw, Lanarkshire ML2 0DP UK; 5 Laboratoire Commun de Biologie et de Génétique Moléculaires, Hôpital Saint-Antoine, 184, rue du Faubourg Saint-Antoine, 75012 Paris, France

## Correction to: BMC Res Notes (2017) 10:487 DOI 10.1186/s13104-017-2811-6

Following publication of the original article [1], the authors requested the following corrections:

1. Author 2—given name should be Dilanthi and family name Warawitage
2. Author 6—given name should be Nalika and family name de Silva

The original article has been corrected.
